# Effect of viscoelastic properties of cellulose nanocrystal/collagen hydrogels on chondrocyte behaviors

**DOI:** 10.3389/fbioe.2022.959409

**Published:** 2022-08-11

**Authors:** Donglei Liu, Hao Zhang, Xufeng Dong, Lin Sang, Min Qi

**Affiliations:** ^1^ School of Basic Medicine, Binzhou Medical University, Yantai, China; ^2^ School of Materials Science and Engineering, Dalian University of Technology, Dalian, China; ^3^ Department of Orthopedics, Central Hospital of Dalian University of Technology, Dalian, China; ^4^ Changchun SinoBiomaterials Co., Ltd., Changchun, China; ^5^ School of Automotive Engineering, Dalian University of Technology, Dalian, China

**Keywords:** hydrogel, viscoelasticity, stress relaxation, creep, chondrocyte behavior

## Abstract

Cartilage tissue engineering technology provides a solution for treating osteoarthritis. Based on the viscoelastic nature of articular cartilage, many viscoelastic hydrogel scaffolds have been developed for investigating the effects on chondrocyte behaviors. However, cellulose nanocrystal/collagen (CNC/COL) hydrogels have not been used as a viscoelastic microenvironment to study chondrocyte growth. Here, we prepared CNC/COL hydrogels with tunable viscoelastic properties and investigated their influences on chondrocyte behaviors. The results showed that CNC and COL within the hydrogels are bonded by hydrogen bonds. The hydrogels had a microporous structure, and the viscoelastic properties were enhanced by increasing the concentration of CNC. Moreover, enhancing the hydrogel viscoelastic properties, including stress relaxation, creep, storage modulus, and loss modulus, promoted the cell shape change, proliferation, and matrix deposition and reduced the IL-1β level. Using a principal component analysis (PCA), stress relaxation was assessed to have the strongest correlation with chondrocytes behaviors, with an authority weight value of 62.547%. More importantly, FAK and YAP were involved in the chondrocytes’ response to the rapid relaxing hydrogel by immunofluorescence staining.

## Introduction

Osteoarthritis is a common chronic disease characterized by degeneration of articular cartilage (Getgood et al., 2009). In addition, articular cartilage does not have blood vessels, nerves, and lymphatics, and it has a limited intrinsic healing capacity (Klein et al., 2010). Thus, osteoarthritis can lead to severe disability in patients if it is not treated seriously. Cartilage tissue engineering technology provides a solution for repairing cartilage defects (Lee et al., 2007). The growth status of chondrocytes in hydrogel scaffolds largely determines the quality of resulting cartilage tissue (Mauck et al., 2000; Lima et al., 2007). Therefore, it is of great scientific significance to study the chondrocyte behaviors at the cellular level for repairing damaged cartilage.

Over the past decade, it has been demonstrated that hydrogels can serve as synthetic extracellular matrix (ECM) microenvironments and capture some features of ECM such as mechanical cues to modulate cellular behaviors significantly. Many studies have shown the correlation of stiffness with cellular behaviors such as cell adhesion, morphology, mobility, and disease progression (Discher et al., 2005; Engler et al., 2006; Solon et al., 2007; Walker et al., 2021). Walker et al. (2021) showed that nuclear mechano-sensing drove distinct chromatin signatures in persistently activated fibroblasts cultured on hydrogels with increased stiffness, which is of great significance to the study of fibrosis. To provide material platform for these studies, elastic polyacrylamide hydrogels are commonly used, but unfortunately it do not exhibit the same time-dependent and viscoelastic responses as biological tissues (Lee et al., 2019). Moreover, Bauer et al. (2017) found that elastic stress in hydrogels restricted some normal cellular process, such as shape change, proliferation, and matrix deposition.

In recent years, an increasing number of literatures have shown that hydrogels with viscoelastic properties are capable of accelerating cellular processes in comparison with stiffness. Researchers have developed various types of viscoelastic hydrogels and elucidated the interaction of cells with two-dimensional (2D) or three-dimensional (3D) hydrogels with different viscoelastic levels (Cameron et al., 2014; Chaudhuri et al., 2016; Bauer et al., 2017; Lee et al., 2017; Chester et al., 2018; Lou et al., 2018; Richardson et al., 2018). Bauer et al. (2017) prepared a set of 2D alginate hydrogels with varying initial elastic moduli and stress relaxation rates. They found that stress-relaxing hydrogel promoted myoblasts spreading and proliferation compared to the elastic with the same stiffness. With the development of highly controllable viscoelastic hydrogels and the deepening understanding of cell–ECM interactions, many works have made important advances in hydrogel viscoelasticity regulating cellular behaviors. For example, Cameron et al. (2014) found that higher loss modulus of 2D collagen/polyacrylamide hydrogel encouraged the mesenchymal stem cells (MSCs) proliferation, spread, and differentiation potential toward a smooth muscle cell (SMC) lineage. The creep-induced loss of cytoskeletal tension resulted in the increase in spread area and the increased Rac 1 activity transformed MSCs into a SMC lineage. In addition, Chester et al. (2018) showed that the difference in loss tangent of N-isopropylacrylamide microgel films could regulate fibroblast migration modes. Interestingly, it reported that faster stress relaxation of 3D alginate hydrogels enhanced fibroblast spreading, proliferation, and osteogenic differentiation of MSCs (Chaudhuri et al., 2016). The arginine–glycine–aspartic acid (RGD) ligands locally clustering, actomyosin contractility, Rho signaling pathway, and YAP nuclear localization were demonstrated to be involved in cellular responses to altered hydrogel stress relaxation. Similarly, faster stress relaxation in the hyaluronic acid/collagen hydrogels promoted cell spreading, fiber remodeling, and focal adhesion (FA) formation in 3D culture (Lou et al., 2018). Notably, Lee et al. (2017) found that faster relaxation and creep and greater loss tangent of alginate hydrogels promoted cartilage matrix formation and proposed that the chondrocytes sensed the substrate mechanical properties by cell volume confinement in 3D culture. Furthermore, hydrazone cross-linked poly(ethylene glycol) hydrogels with tunable stress relaxation times from hours to months were formulated by Richardson et al. (2018). Moreover, 4 weeks post-encapsulation of chondrocytes, the hydrogel with relaxation times of 3 days enhanced the cellularity and cartilage matrix interconnectivity.

As mentioned before, various hydrogel materials are utilized to show that cellular processes associated with their viscoelastic properties have important implications for repairing cartilage defects. However, the CNC/COL hydrogels with different viscoelastic properties have not been used to study chondrocyte behaviors. Here, we used microporous CNC/COL hydrogels as a tunable material platform for investigating the role of the viscoelastic properties in chondrocyte behaviors and elucidating the related intracellular mechano-transduction mechanism. Collagen, a natural protein, possesses inherent cytocompatibility due to the presence of cell adhesion ligand binding sites, but low Young’s modulus limits its development (Zhang et al., 2014; Yang et al., 2019). To address this issue, a common strategy is to utilize composite technology to add nanoscale reinforcement additive into collagen. CNC is a kind of renewable rod-shaped nanocrystals. It possesses many desirable properties, such as no cytotoxicity, high strength, stiffness, excellent hydrophilicity, and water retention capacity due to the abundance of surface hydroxyl groups (Martha et al., 2014; Trache et al., 2017; Abitbol et al., 2018; Biswal et al., 2020). Moreover, the CNC/COL hydrogels have a wide range of viscoelastic properties by changing component concentration (Liu et al., 2020). Since the stiffness (represented by storage modulus) of the CNC/COL hydrogels is not independent, the authority weights of viscoelastic parameters such as stress relaxation, creep, storage modulus, and loss modulus on the behavior of chondrocytes were assessed using PCA by SPSS software.

## Materials and methods

### Materials

Cellulose microcrystal (CMC) was purchased from Hainan Yide Food Co., Ltd. (Hainan, China). Rat tail type I collagen (COL, 3 mg/ml) was bought from GIBCO Invitrogen Corporation (California, United States). Acetic acid, ethanol, N-(3-Dimethylaminopropyl)-N′-ethylcarbodiimide hydrochloride (EDAC), and N-Hydroxysuccinimide (NHS) were purchased from Aladdin (Shanghai, China). The deionized water was provided by our laboratory. Primary chondrocytes were provided by Beijing Baiou Bowei Biotechnology Co., Ltd. (Beijing, China), and the cells were verified to be free of mycoplasma by the manufacturer. Dulbecco’s modified Eagle’s medium (DMEM), fetal bovine serum (FBS), and penicillin/streptomycin were bought from GIBCO Invitrogen Corporation (California, United States). L-ascorbic acid-2-phosphate was purchased from Sigma (Shanghai, China). Trypsin/EDTA solution, paraformaldehyde, sucrose, optimal cutting temperature compound (OCT), Triton X-100, goat serum (Solarbio), DAPI, Alexafluor 488 phalloidin, and papainase were bought from Beijing Solarbio Science & Technology Co., Ltd. (Beijing, China). Rabbit anti-collagen II polyclonal antibody (bs-10589R), rabbit anti-ACAN polyclonal antibody (bs-1223R), and goat anti-rabbit IgG/Cy3 (Cat. #bs-0295G-Cy3) were purchased from Beijing Biosynthesis Biotechnology Co., Ltd. (Beijing, China). FAK (focal adhesion kinase) rabbit mAb (Cat. #A11131) and YAP1 (Yes associated protein 1) rabbit pAb (Cat. #A1002) were bought from Wuhan ABclonal Biotechnology Co., Ltd. (Wuhan, China). PicoGreen assay, hydroxyproline assay, 1,9-dimethylmethylene blue (DMMB) assay, lambda phage DNA, chondroitin sulfate, and L-hydroxyproline were purchased from Genmed Scientifics Inc., United States (Shanghai, China). The rat IL-1β ELISA reagent kit and recombinant rat IL-1β were bought from Jiangsu MEIMIAN Industrial Co., Ltd. (Jiangsu, China).

### Cellulose nanocrystal preparation

The CNC was prepared using the homogenization method (Liu et al., 2021). First, the 0.2 wt.% CMCs aqueous solution was sealed with a plastic wrap and swelled for 24 h at room temperature. Then the obtained CMC gel was sheared at 12,000 rpm for 5 min and centrifuged at 8,000 rpm for 20 min, and then the clear phase was collected. Finally, the collected CNCs dispersion was used for freeze-drying before use.

### Cellulose nanocrystal/collagen hydrogel preparation

The CNC/COL hydrogels with different viscoelastic levels were prepared as described previously (Liu et al., 2021). In brief, COL was blended with 0.5 M acetic acid for 90 min in a cooled reaction vessel using a homogenizer at a speed of 10,000 rpm for 5 min. Cellulose nanocrystals (CNCs) were added in aliquots to the collagen solution every hour during blending to obtain the mixed solutions with a final collagen concentration of 0.5 wt.% and varying CNC concentrations of 2.5 wt.%, 5 wt.%, 10 wt.%, and 15 wt.%, respectively. The mixtures were degassed in a vacuum desiccator for 60 min. They were poured in a culture dish and froze for 4 h at −35°C. Then they were freeze-dried at −40°C and 10 Pa for 72 h to obtain dehydrated porous samples. Next, the samples were cross-linked in an ethanol solution of EDAC and NHS using a concentration of 6 mM EDAC g^−1^ of collagen, and a 5:2 M ratio of EDAC:NHS at 37°C for 72 h under oscillation, after which they were rinsed several times with deionized water. Finally, the hydrogel samples were punched into cylinders (6 mm diameter × 3 mm height) for using in following experiments.

### Atomic force microscopy characterization

The CNCs height dimensions were evaluated by atomic force microscopy (AFM, Bruker Dimension 3100 SPM) in the tapping mode. Rectangular ferromagnetic resonance cantilevers with a spring constant of 2.4 N/m and a resonance frequency of 60 kHz were used to image CNCs on the mica substrates. The data was obtained in three different positions on three different images. All the measurements were done in air at room temperature.

### Fourier transform infrared spectrometer characterization

The functional groups of the freeze-dried pure collagen hydrogel, CNCs dispersion, and CNC/COL hydrogels with different CNC concentrations were analyzed by using the Fourier transform infrared spectrometer (FTIR, Thermo Fisher Scientific, Nicolet 6700). A diamond crystal plate was used and the scanning wavelength ranged between 500 and 4,000 cm^−1^, with a resolution of 4 cm^−1^, and the number of scanning speed was 30 s^−1^.

### Scanning electron microscope characterization

The microstructures of the CNC/COL porous scaffolds were observed using a field-emission scanning electron microscope (SEM, Zeiss, SUPARR-55) at an acceleration voltage of 5 kV. The cross sections of the CNC/COL porous scaffolds were coated with gold by sputtering at 30 mA for 40 s using a sputter coater (Quorum, Q150V ES) before observation. The pore size of scaffolds was measured manually by evaluating three SEM images of three samples but with the same CNC concentration using ImageJ software (Bethesda). The detailed process includes importing the SEM image, setting scale, and measuring pore size. The results of pore size were statistically analyzed by using Origin 8.0 software (Origin Lab).

### Rheological characterization

The viscoelastic properties of CNC/COL hydrogels were measured using a rotational rheometer (Anton Paar, Physica MCR 301). The hydrogel was submerged in water at 37°C during the tests. Cylindrical hydrogel was placed between parallel plates and the gap between the plates, which was adjusted using a normal force of 0.1 N in order to prevent slippage. All measurements did not run until the hydrogel relaxed to an equilibrium state. In stress relaxation test, a shear strain of 100% was applied on the hydrogel and maintained constant up to the end of the test while recording the corresponding shear stress, as a function of time. A creep test was performed by applying a constant shear stress of 100 Pa on the hydrogel and recording the shear strain over time, followed by creep recovery. A strain sweep was carried out at a frequency of 1 Hz with amplitudes ranging between 0.01% and 100% to measure the storage modulus and loss modulus as functions of strain amplitude. Each test was repeated three times.

### Primary chondrocyte culture

The culture of primary chondrocytes was conducted according to a previously published method (Lou et al., 2018). In brief, the chondrocytes isolated from rat cartilage were cultured in 75 cm^2^ tissue culture flasks (Thermo Fisher Scientific) in standard DMEM with an atmosphere of 5% CO_2_ at 37°C. The DMEM was supplemented with 10% FBS, 0.05 mg ml^−1^ L-ascorbic acid-2-phosphate, and 1% penicillin/streptomycin. The cell culture medium was refreshed every 3 days.

### Seeding chondrocytes into porous hydrogels

For cell culture use, the lyophilized hydrogels were sterilized with 70% ethanol, washed three times with deionized water, and conditioned with DMEM at 37°C for 60 min. The chondrocytes were harvested by treatment with a 0.05% trypsin/ethylene diamine tetra acetic acid solution when the cells reached a confluence of 80%. The harvested chondrocytes (P1 chondrocytes) were re-suspended in DMEM to prepare a cell suspension solution of 1 × 10^5^ cells ml^−1^ for cell seeding. The concentration of the cells was determined using a Coulter counter (Beckman Coulter). The P1 chondrocytes were seeded into the hydrogels by adding 1 ml of the cell suspension solution (1 × 10^5^ cells per hydrogel) to each of the cylindrical sides of the hydrogels.

### Immunohistochemistry

The hydrogels containing chondrocytes cultured for 3, 7, and 14 days were fixed with 4% paraformaldehyde for 60 min and washed three times in phosphate-buffered saline (PBS). The hydrogels were placed in 30% sucrose at 4°C for 1 day and then incubated in optimal cutting temperature compound (OCT)–sucrose, a mixture of 50% OCT and 30% sucrose solution, for 5 h. They were then embedded in OCT, frozen, and sectioned. The sections were prepared with a thickness of approximately 50 μm using a cryostat (Leica CM1950) and processed using standard immunohistochemistry procedures. Sections were washed three times in PBS, permeabilized with PBS containing 0.5% Triton X-100 for 20 min, and blocked with a blocking buffer composed of 10% goat serum and 0.1% Triton X-100 in PBS for 30 min. The following antibodies and reagents were used for immunostaining: DAPI, Alexafluor 488 Phalloidin, rabbit anti-collagen II polyclonal antibody, rabbit anti-ACAN polyclonal antibody, goat anti-rabbit IgG/Cy3, FAK rabbit mAb, and YAP1 rabbit pAb. Each immunostaining was performed in six sections obtained from three hydrogels with the same CNC concentration.

### Biochemical analysis

In brief, the hydrogels containing chondrocytes was cultured for 14 days were freeze-dried and their dry mass was measured. The dried samples were then digested in PBS with papainase at 60°C for 16 h. The PicoGreen assay was conducted to measure the amount of DNA in the hydrogels. Lambda phage DNA was used as the standard for DNA quantity under the assay. The proliferation rate was calculated by the measured DNA amounts divided by the DNA amounts of seeded cells. The DMMB assay was applied to measure the amounts of GAGs in the hydrogels. Chondroitin sulfate was used as the standard of GAGs amount with the DMMB assay. The hydroxyproline assay was utilized to measure the amount of the collagen matrix in the hydrogels. L-Hydroxyproline was used as the standard of hydroxyproline amount under the assay. The collagen amount was calculated by a mass ratio of hydroxyproline: collagen of 1:7.46. The measured values of collagen, GAGs, and DNA amounts were normalized with the dry mass of the hydrogels. Three samples were used for each biochemical analysis.

### IL-1β level analysis

The hydrogels containing chondrocytes were removed from culture medium after 14 days of culture and washed in PBS. They were crushed with pestles for homogenization. The crushed samples were then totally disassociated by adding 2 mM EDTA solution. The suspension was centrifuged at 10,000 r.p.m. for 2 min and the supernatant was extracted. The rat IL-1β ELISA reagent kit was utilized to quantify the protein concentration of IL-1β was quantified, and the recombinant rat IL-1β was used as a standard. The values were normalized to DNA amounts in each of the hydrogels. Three samples were used for each analysis.

### Image analysis

For assessing spreading, immunohistochemical staining of the hydrogel sections containing chondrocytes for DAPI/phalloidin were imaged using an inverted fluorescence microscope (Olympus, lx71). The DAPI channel was used for nuclei detection and the phalloidin channel was used for cell body detection. The area of the chondrocytes was determined using a custom routine (threshold–analyze particles) in ImageJ. The aspect ratio of the chondrocytes was measured manually by ImageJ. The cell area and aspect ratio values were obtained from four phalloidin staining images, respectively. For measurements of FAK localization in the chondrocytes, DAPI/phalloidin/FAK antibody staining of hydrogel sections was imaged. For measurements of YAP localization, DAPI/phalloidin/YAP antibody staining of hydrogel sections was imaged.

### Principal component analysis

Utilizing IBM SPSS Statistics 20.0 software, principal component analysis (PCA) was used to assess the authority weight of the viscoelastic parameters, including stress relaxation time (*τ*_1/2_), creep time (*γ*_1/2_), storage modulus (*G*′) and loss modulus (*G*″), in study of chondrocyte behaviors. Specifically, the data on spreading, proliferation, and matrix levels were all imported into the workbook to obtain the weight values.

### Statistical analysis

Statistical differences were determined by analysis of variance or Student’s *t* test where appropriate, with significance indicated by *p* < 0.05.

## Results

### Atomic force microscopy analysis

**FIGURE 1:**
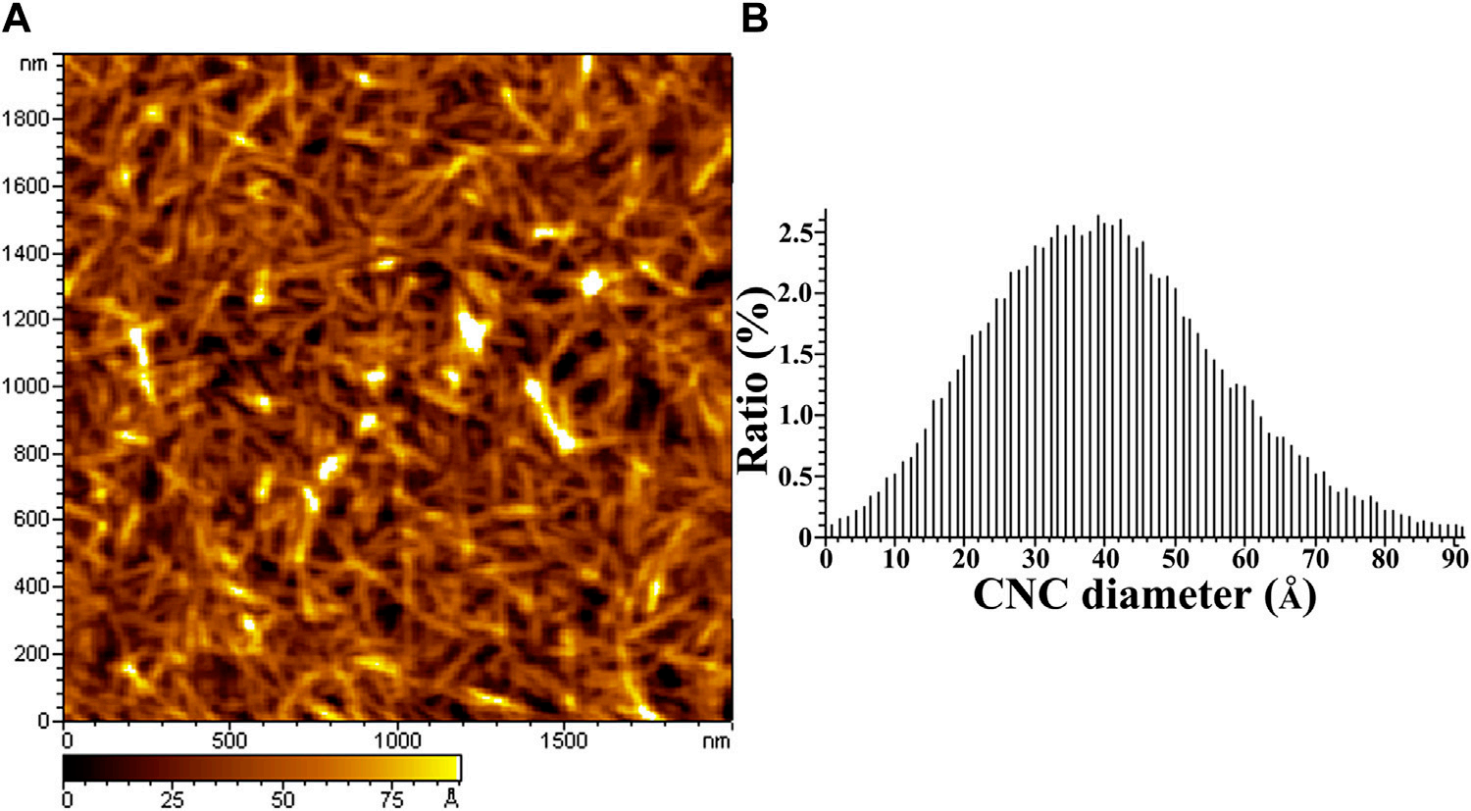
AFM height image and the diameter distribution of CNCs. **(A)** is the AFM image of CNCs, **(B)** is the diameter distribution of CNCs.

Figure 1 shows the AFM image of CNC (Figure 1A) and its corresponding AFM measured height distribution (Figure 1B). CNC with length of several hundred nanometers is visible from the AFM image and the average diameter of 4 nm is found in the height statistics of the image.

### Fourier transform infrared spectrometer analysis

**FIGURE 2:**
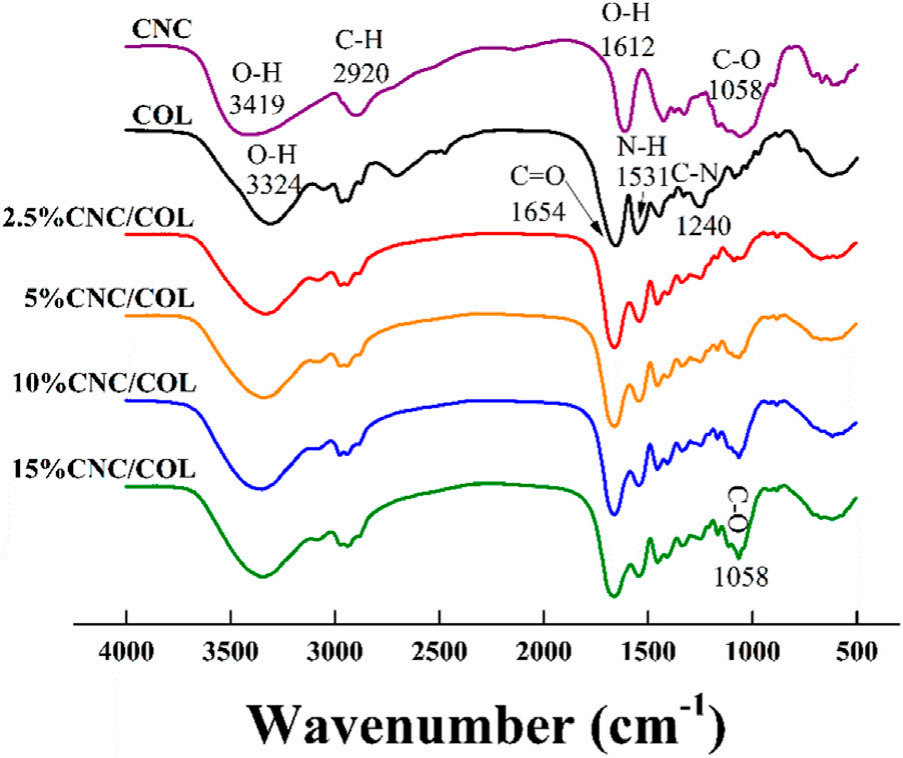
FTIR spectrogram of CNC/COL hydrogels with different CNC concentrations.

As shown in Figure 2, some characteristic absorptions of CNC occur at 3,419, 2,920, 1,612, and 1,058 cm^−1^, corresponding to stretching vibrations of O-H, C-H, O-H, and C-O (Martha et al., 2014; Jayaramudu et al., 2018; Jayaramudu et al., 2019; Liu et al., 2020; Liu et al., 2021). For collagen hydrogel (COL), the absorptions at 3,324, 1,654, 1,531, and 1,240 cm^−1^, can be ascribed to stretching vibrations of the O-H in amide I, the C=O bond in amid, the N-H and C-H bonds in amide II, and the C-N bond in amide III, respectively (Zhang et al., 2014; Yang et al., 2019). Notably, the absorption peak at 3,324 cm^−1^ occurs a slight blue shift with the addition of CNC to the collagen hydrogel, which indicates that the two materials are bonded by hydrogen binding (Jayaramudu et al., 2018; Jayaramudu et al., 2019). Further, the absorption that is at 1,058 cm^−1^ becomes strong gradually with CNC concentration, which further indicates that CNC has been successfully introduced into the hydrogels.

### Scanning electron microscope analysis

**FIGURE 3:**
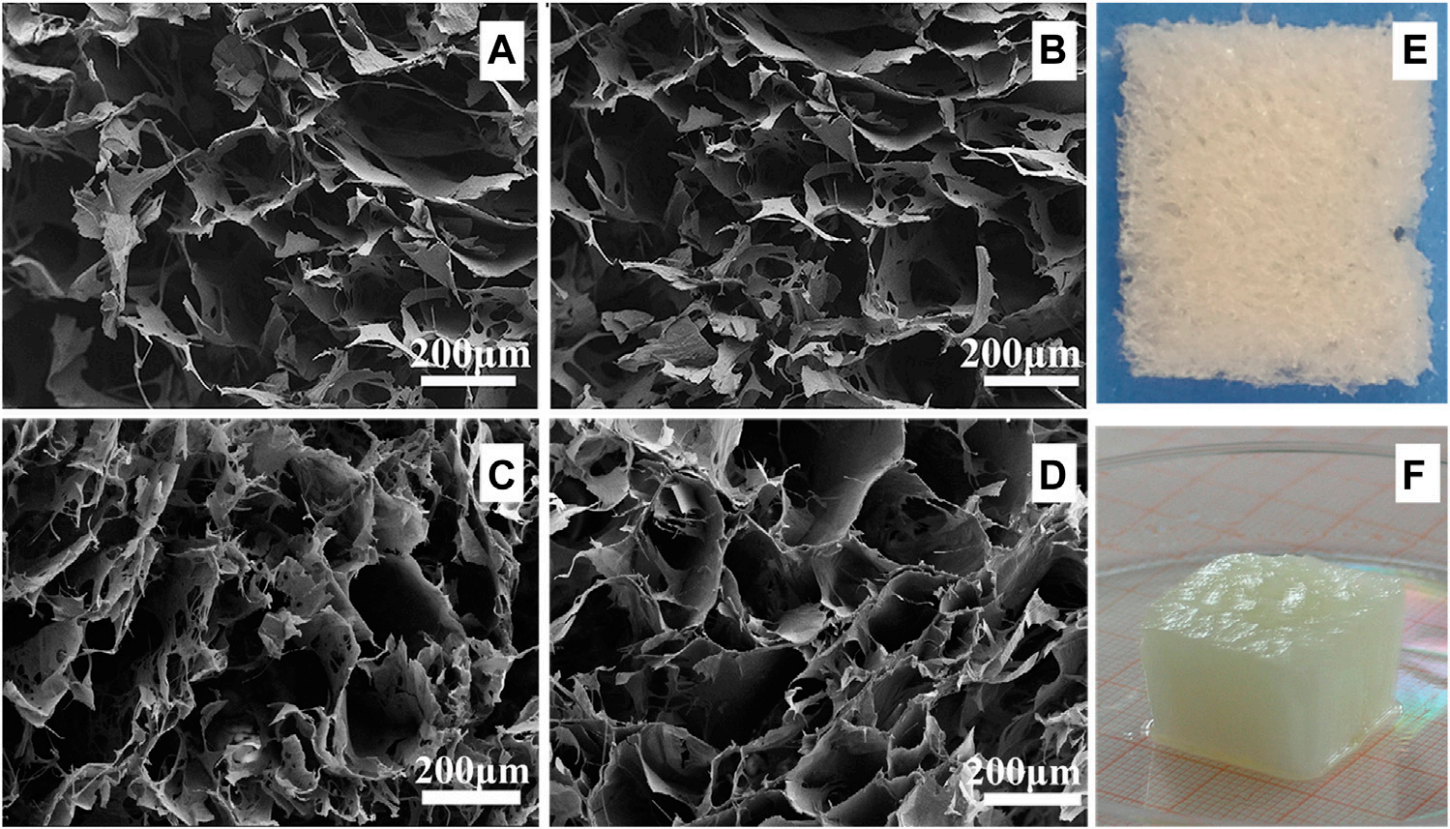
SEM images and digital photographs of CNC/COL hydrogels with different CNC concentrations. **(A)** 2.5 wt.% CNC, **(B)** 5 wt.% CNC, **(C)** 10 wt.% CNC, and **(D)** 15 wt.% CNC; **(E)** and **(F)** are the digital photographs of the cryogel and the hydrogel with 15 wt.% CNC.

As shown in Figure 3, the hydrogels with varying CNC concentrations display a microporous structure. The pore size is 222 ± 29 μm, 214 ± 19 μm, 202 ± 17 μm, and 195 ± 20 μm under the CNC concentration of 2.5 wt.%, 5 wt.%, 10 wt.%, and 15 wt.%. The pore size of the hydrogel with 2.5 wt.% CNC is significantly larger than that of the hydrogel with 15 wt.% CNC (independent samples t test, *p* < 0.05). The range of pore size falls within the optimal pore diameter range of 150–250 μm, which is reported to promote the expression and production of type II collagen and aggrecan in culture of chondrocytes (Zhang et al., 2014; Andrea et al., 2018).

### The viscoelastic properties of cellulose nanocrystal/collagen hydrogels

**FIGURE 4:**
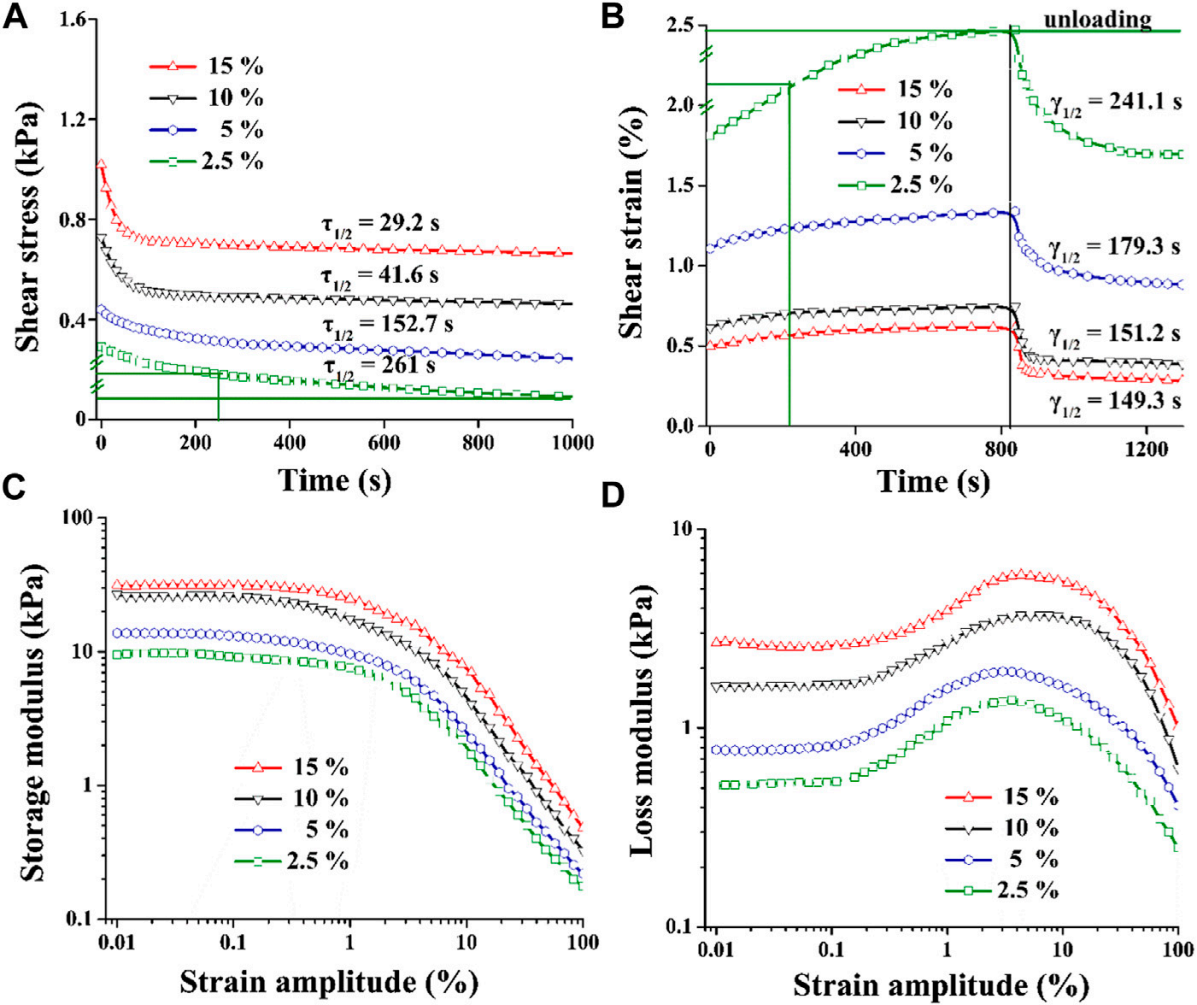
Viscoelastic properties of CNC/COL hydrogels with varying CNC concentrations. **(A)** stress relaxation, **(B)** creep, **(C)** storage modulus, and **(D)** loss modulus.

Previous studies have demonstrated that the 3D microporous CNC/COL hydrogels displayed a wide and tuneable range of viscoelastic properties (Matellan and Hernandez, 2019). This is confirmed, as it is found that by increasing CNC concentration from 2.5% to 15%, the rate of stress relaxation (*τ*_1/2_) and creep (*γ*_1/2_), and the value of *G*’ and *G*’’ are enhanced markedly (Figure 4). *τ*_1/2_ is defined as the quantification of timescale at which the shear stress relaxes to half its total decreased value. *γ*_1/2_ is the quantification of timescale at which the shear strain goes up to half its total increased value. Specifically, the *τ*_1/2_ value of the hydrogels ranges from ∼29.2 to ∼ 261 s, and the frequency at which the strain sweep measures *G*′ and *G*″ is 1 Hz (Figure 4A). These timescales are related to some cellular behaviors, as cells are thought to exert traction with a timescale of minutes and respond to oscillating forces over a timescale of ∼1 s (Mow et al., 1991). In addition, the *G*′ value of the hydrogels ranges from 10 to 30 kPa in the linear viscoelastic region (Figure 4C), and it is an order of magnitude smaller than cartilage ECM which has a shear modulus of 200 ∼ 500 kPa (Cameron et al., 2011; Loebel et al., 2020).

### Chondrocyte spreading and proliferation

**FIGURE 5:**
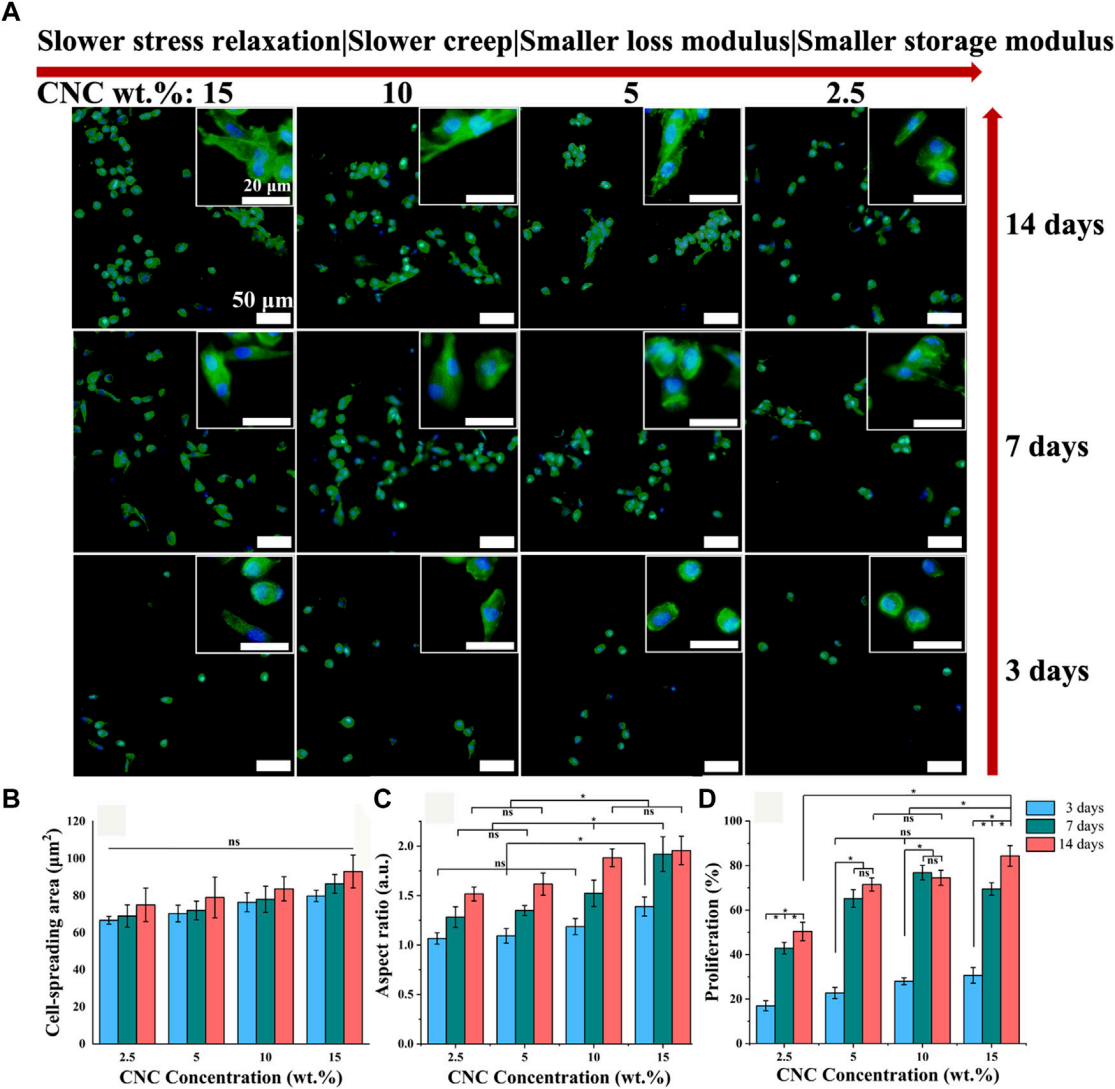
Cell spreading and proliferation for chondrocytes cultured in hydrogels with different viscoelastic properties. **(A)** Images of chondrocytes cultured in hydrogels with the indicated CNC concentration (2.5 wt.%, 5 wt.%, 10 wt.%, and 15 wt.%). Green represents actin staining and blue represents nucleus. Images were taken after 3, 7, and 14 days in culture. Scale bar is 50 μm. **(B–D)**, Spreading area, aspect ratio, and proliferation of chondrocytes (*p < 0.05).

The microporous series of viscoelastic CNC/COL hydrogel scaffolds were used to culture the chondrocytes, and the effects of hydrogel viscoelasticity on cell spreading and proliferation behaviors were firstly studied. Figure 5A shows the immunofluorescence staining images of nucleus and actin skeleton of chondrocytes. After culture for 3 days, some chondrocytes in the hydrogels with higher viscoelastic properties (10% and 15% CNC), show elongated shape, while the chondrocytes in the hydrogels with lower viscoelastic properties (2.5% and 5% CNC), remain spherical shape, which is similar to the typical morphology when cell growth is inhibited in elastic hydrogel (Richardson et al., 2018). The variance in cell shape indicates that increasing hydrogel viscoelasticity can promote the morphological transformation of chondrocytes. With the prolonging of culture time (3–14 days), the chondrocytes in the hydrogels with the same viscoelastic level all show a tendency to spindle shape from spherical shape. However, at lower viscoelastic levels (2.5% and 5% CNC), the chondrocytes don’t show significant shape change until 14 days of culture. In addition, the chondrocytes under the lowest viscoelastic level (2.5% CNC) emerge a spindle shaped change, but the number of cells is much lower than that under the other three viscoelastic levels (5%, 10%, and 15% CNC). These results suggest that increasing the rate of stress relaxation and creep, as well as storage modulus and loss modulus of CNC/COL hydrogels can promote chondrocyte growth.

Judging by the morphological transformation of chondrocytes, the process of cell spreading is accompanied by constantly exerting traction on the surface of hydrogel pores (Mow et al., 1991). It implies that the CNC/COL network under the pore surface is reconstructed dynamically by the chondrocytes. The mechanical reconstruction of CNC/COL network in turn provides larger space for cell spreading (Chaudhuri et al., 2016; Chester et al., 2018). Thus, the faster stress relaxation or creep of hydrogels will result in the higher aspect ratio of chondrocytes.

For this, the spreading and proliferation of chondrocytes cultured in the hydrogels with different viscoelastic levels are measured, and the results are shown in Figures 5B–D. With the prolonging of culture time and the increasing of viscoelastic properties, there is no significant difference in the spread area of chondrocytes, but its aspect ratio shows an increasing trend (*p < 0.05). At 3 days of culture, the cell aspect ratio in the hydrogel with the highest viscoelastic level (15% CNC) is significantly bigger than that in the other three viscoelastic hydrogels (*p < 0.05), which is consistent with the cell morphology observed in Figure 5A. At 7 and 14 days of culture, the aspect ratio in the hydrogels with higher viscoelastic levels (10% and 15% CNC) is still bigger than that in the hydrogels with lower viscoelastic levels (2.5% and 5% CNC). In addition, the proliferation rate of chondrocytes in no matter what kind of viscoelastic hydrogels presents stepped increase from 3 to 7 days of culture (Figure 5D, *p < 0.05). Further, the cell proliferation in the highest viscoelastic hydrogel is larger than that in the other three viscoelastic hydrogels at 14 days of culture (*p < 0.05). These suggest that rapid stress relaxation and creep, as well as high storage modulus and loss modulus can promote chondrocytes growth such as elongation and proliferation.

### Cartilage matrix formation

**FIGURE 6:**
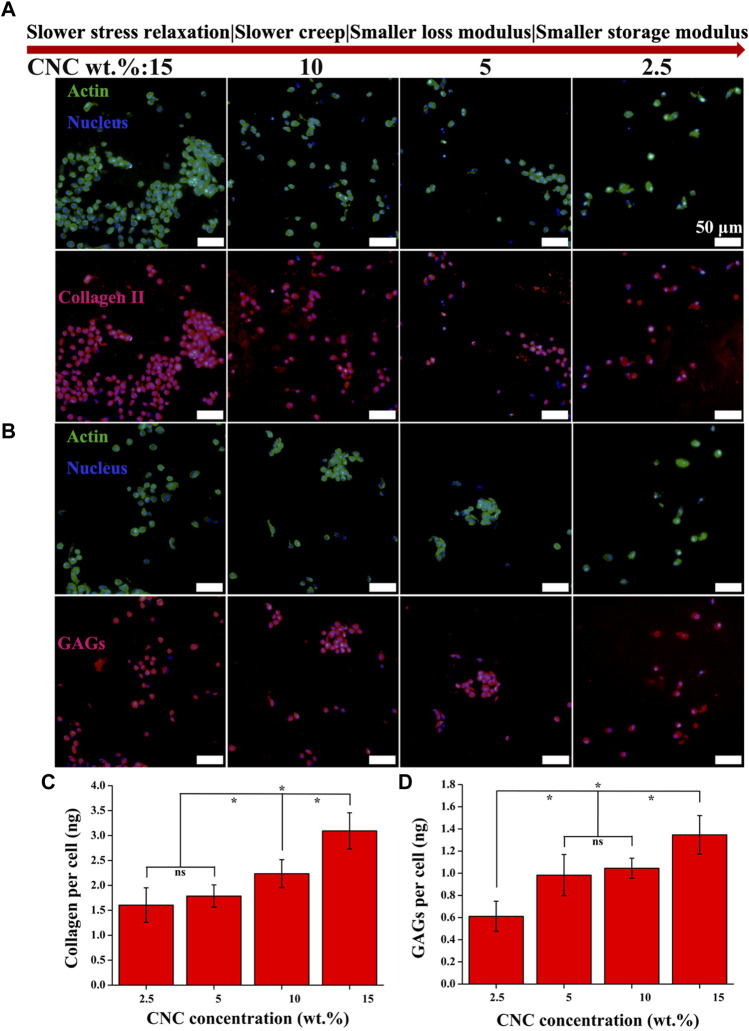
COL II and GAGs productions by chondrocytes cultured in the hydrogels with varying viscoelastic levels. **(A,B)** Images of COL II and GAGs staining for chondrocytes after 14 days of culture. **(C,D)** Quantification of the accumulated COL II and GAGs for chondrocytes after 14 days of culture (*p < 0.05).

Type II collagen (COL II) and aggrecan (GAGs), critical components of cartilage matrix secreted by chondrocytes, were assessed with immunohistochemical staining after 14 days of culture. As shown in Figures 6A,B, both COL II and GAGs are deposited in the region adjacent to the chondrocytes, not connected together, which is mainly attributed to the low viability of chondrocytes (Burdick and Prestwich, 2011; Loebel et al., 2020; Patel et al., 2021). With the increase of stress relaxation and creep rate, as well as storage modulus and loss modulus, the total area of COL II formation is observed to show an obvious increasing trend relative to GAGs. Further, as the hydrogels were cut into sections, greater area of cartilage matrix in the sections corresponds to greater volume of matrix in the hydrogels, i.e., the higher level of extracellular matrix secreted by the chondrocytes. To verify the inference, colorimetric method for hydroxyproline and DMMB content was used to quantitatively assess the amounts of both COL II and GAGs in the hydrogels with different viscoelastic levels. As shown in Figures 6C,D, the higher levels of both COL II and GAGs are measured in the hydrogels with faster stress relaxation and creep, as well as higher storage modulus and loss modulus (*p < 0.05). In the highest viscoelastic hydrogel (15% CNC), the level of COL II (∼3 ng/cell) is 2-fold higher than that of GAGs (∼1.4 ng/cell).

### Immunofluorescent staining of focal adhesion kinase and yes associated protein

**FIGURE 7:**
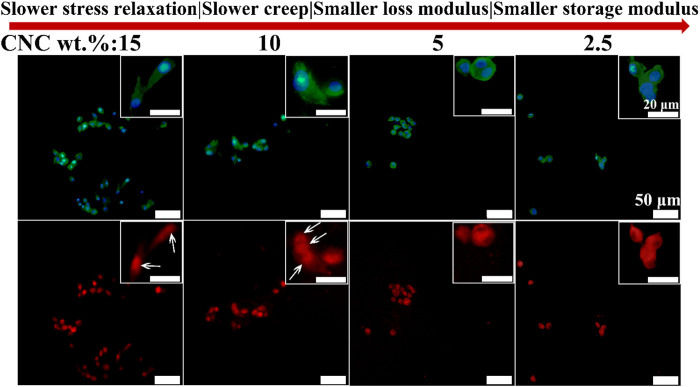
FAK staining of chondrocytes cultured in different viscoelastic hydrogels. Green represents actin staining, blue represents nucleus staining, and red represents FAK staining. Images were taken after 14 days in culture. White arrow indicates the location of FAK.

Next, the roles of Focal Adhesion Kinase (FAK) and Yes Associated Protein (YAP) in the effect of viscoelastic properties of hydrogels on the chondrocyte behavior were investigated. FAK has been identified as a regulator in cell adhesion, migration and survival, and a critical signal transducer for cell–ECM mechanical interactions (Mitra and Schlaepfer, 2006; Brusatin et al., 2018). Figure 7 shows the immunofluorescence staining images of FAK in the chondrocytes cultured in the hydrogels with different viscoelastic levels. It can be found that FAK presents a dispersive distribution throughout the cytoplasmic region outside the nucleus in the chondrocytes cultured in the lowest viscoelastic hydrogels (2.5% CNC). As CNC concentration increases from 2.5% to 5%, FAK tends to accumulate in the cytoplasm. At the CNC concentration of 10%, FAK is observed to aggregate in the cytoplasm. As the CNC concentration increases to 15%, FAK is concentrated and localized on one side of the nucleus in some chondrocytes. These results indicate that increasing the rate of stress relaxation and creep, storage modulus and loss modulus of the hydrogels, can promote FAK localizing around the nucleus of chondrocytes.

**FIGURE 8:**
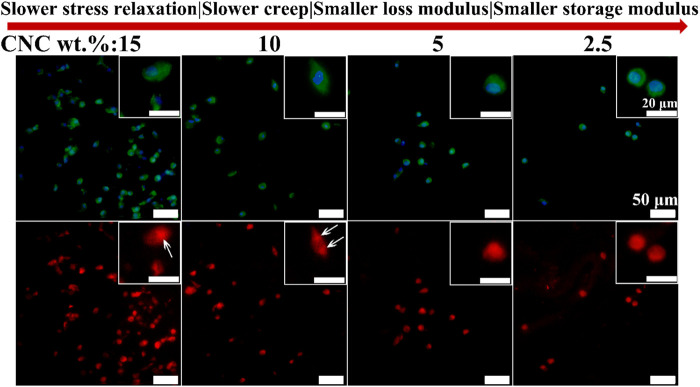
YAP staining of chondrocytes cultured in different viscoelastic hydrogels. Green represents actin staining, blue represents nucleus, and red represents YAP. White arrow indicates the location of YAP.

YAP is known as a mechanosensitive transcription factor that is the key regulatory element in controlling the gene expression of cells response to mechanical cues from ECM (Daheshia and Yao, 2008; Sirio et al., 2011). Further, it is reported that FAK controlled translocation and activation of YAP in response to mechanical activation (Lachowski et al., 2017). However, the role of YAP in the chondrocyte growth under the viscoelastic stimuli has not been studied. Figure 8 shows the immunofluorescence staining images of YAP in the chondrocytes cultured in different viscoelastic hydrogels. It can be found that YAP is dispersed and distributed throughout the chondrocytes cultured in the lower viscoelastic hydrogels (2.5% and 5% CNC). With increasing the viscoelastic properties, YAP is accumulated on both sides of the nucleus (10% CNC). In the high viscoelastic hydrogel (15% CNC), YAP is found to be localized on one side of the nucleus. These results suggest that faster stress relaxation and creep, higher storage modulus and loss modulus, can enhance the perinuclear translocation of YAP in the chondrocytes.

### Secretion of IL-1β

**FIGURE 9:**
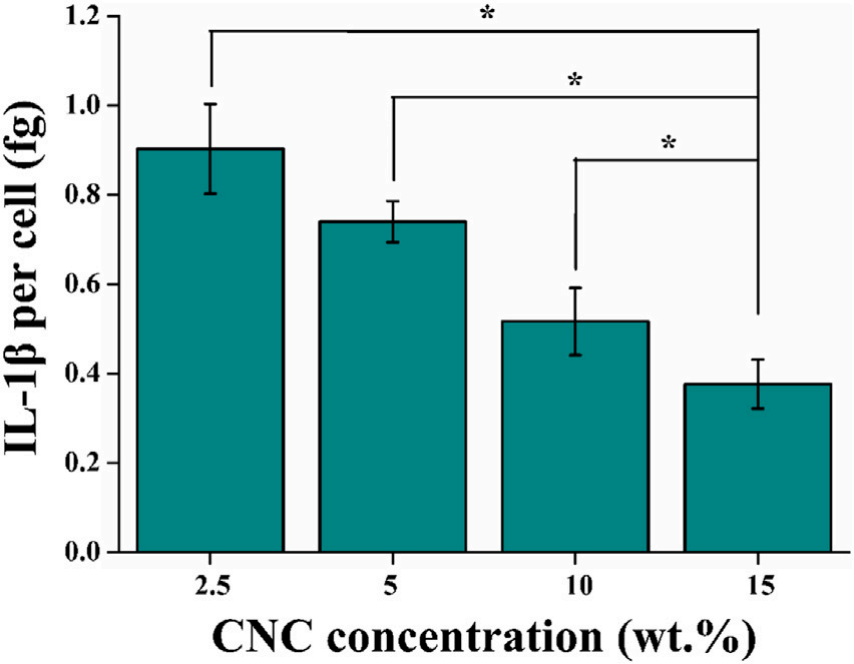
Quantification of the amount of IL-1β secreted into the different viscoelastic hydrogels after 14 days of culture (*p < 0.05).

Finally, the secretion of cytokine interleukin-1β (IL-1β) in different viscoelastic hydrogels were studied. IL-1β, as a major driver of osteoarthritis progression, is recognized to induce chondrocytes apoptosis in osteoarthritic cartilage (Pelletier et al., 1993). As shown in Figure 9, the level of IL-1β protein is decreased in the hydrogels with increasing the viscoelastic properties after 14 days of culture. It implies that the expression of IL-1β is related to the CNC/COL hydrogel viscoelastic properties. This matches the result previously reported in fast relaxing alginate hydrogels (Richardson et al., 2018). Thereby we speculate that the significant difference in proliferation of chondrocytes in the different viscoelastic hydrogels at 3, 7, and 14 days of culture, is primarily due to the varying levels of IL-1β secreted by chondrocytes.

## Discussion

This work exhibits the role of CNC/COL hydrogel viscoelasticity in regulating chondrocytes behavior. It is worth noting that although the prepared hydrogel has a 3D porous structure, its pore size (∼200 μm) is much larger than the size of chondrocytes (∼20 μm) according to the SEM and chondrocyte spreading results, so the microenvironment in which the chondrocytes grew can actually be regarded as a 2D plane structure. Interestingly, the timescale of stress relaxation of the high viscoelastic hydrogel of ∼29.2 s is close to that of cartilage, chondron, and chondrocytes which exhibit viscoelastic responses with a characteristic timescale of ∼10 s (Figure 4) (Richardson et al., 2018). This indicates that the hydrogel may more closely mimic the viscoelasticity of native cartilage microenvironment than the other three viscoelastic hydrogels. Recent studies have used alternative material approaches, including changing crosslinking method, modulating crosslinking density, polymer concentration, and molecular weight, to tune stress relaxation, creep, loss modulus, and storage modulus in hydrogels but hold their stiffness constant (Cameron et al., 2014; Chaudhuri et al., 2016; Bauer et al., 2017; Lee et al., 2017; Chester et al., 2018; Lou et al., 2018; Richardson et al., 2018; Lee et al., 2019). The approach described in this paper is simple and conventional so that the viscoelastic CNC/COL hydrogels do not have independent stiffness (represented by *G*′), but it is unfavorable for studying the effects of viscoelastic properties on chondrocyte behaviors clearly. For this, we propose to calculate the authority weight values of these viscoelastic parameters, including τ_1/2_, γ_1/2_, *G*′, and *G*″ using principal component analysis, and to provide quantifiable viscoelastic effects for probing cell–hydrogel substrate interactions (Liu et al., 2017). Table 1 shows the influence authority of each viscoelastic parameter of the CNC/COL hydrogel. The authority weight value of *τ*_1/2_ is 62.547%, that of *γ*_1/2_ is 18.623%, that of *G*″ is 10.806% and that of *G*’ is 8.024%, and thus the influence authority order is *τ*_1/2_ > *γ*_1/2_ > *G*″ > *G*′. This result indicates that stress relaxation has the strongest correlation with chondrocyte behavior, which is consistent with the viewpoints reported in the most literatures (Cameron et al., 2014; Bauer et al., 2017; Chester et al., 2018). In addition, Kaiser–Meyer–Olkin value >0.6 indicates that this method can be used to analyze the quantized data that describe chondrocyte behavior.

**TABLE 1:** Total variance explained by principal component analysis.

Chondrocyte behaviors	Kaiser–Meyer–Olkin	Viscoelastic parameters	Initial eigenvalues total	Squared loadings variance (%)
Spreading area, aspect ratio, proliferation, levels of COL II, sGAG and IL-1β	0.609	*τ*_1/2_	2.192	62.547
		*γ*_1/2_	0.807	18.623
		*G*″	0.664	10.806
		*G*′	0.337	8.024

Chondrocyte adhesion is the basis of cellular response to the viscoelastic CNC/COL hydrogels, as the hydrogels contain rat tail type I collagen. As described above, the results of cell shape change and proliferation indicate that the chondrocytes have adhered to the hydrogel substrates (the surface of pores within the hydrogel) successfully (Figure 5). By enhancing the concentration of CNC, the mobility of collagen chains decreases and the flowability of matrix composed of collagen network and CNCs reduces due to a steric hindrance of short rod-shaped CNCs, so that the time dependence of stress or strain occurred in the hydrogel substrates diminishes under the imposition of a defined deformation or force (applied by an adhered cell), and the behavior of the hydrogel is referred to as “stress relaxation” or “creep” (Lu and Mow, 2008; McKinnon et al., 2013; Chester et al., 2018). As adherent cells begin to exert force or deformation on a viscoelastic substrate, the substrate viscoelasticity may result in cells feeling a decreasing resistive force and an increasing resistive deformation exerted by cells they experience when actively pulling on a substrate (Chester et al., 2018). Therefore, higher aspect ratio of the chondrocytes is observed in the faster-relaxing hydrogels because the force exerted by cells can be relaxed more quickly and thus converting its form into isotonic contraction (Chaudhuri et al., 2016). These changes in resistive force and deformation from substrate due to its viscoelastic feature, would be expected to not only activate or inhibit some signal molecules, but impact many other downstream cellular processes, such as cell spreading, proliferation, and matrix formation.

Cell spreading in turn is known to mechanically activate some signal molecules (Zhang et al., 2014; Lee et al., 2017; Richardson et al., 2018). FAK and YAP play well-known and fundamental roles in cellular mechano-sensing and mechano-transduction (Abitbol et al., 2018). It was reported that activation (phosphorylation) of FAK, a signal transducer in focal adhesions (FAs), which increased actomyosin contractility and drove actin polymerization (Mitra and Schlaepfer, 2006). Actin polymerization in turn modulated the nuclear translocation of YAP directly and started a series of cellular processes (Sirio et al., 2011; Brusatin et al., 2018). Therefore, we speculate that both FAK and YAP signal molecules participate in the growth process of chondrocytes stimulated by CNC/COL hydrogel viscoelasticity. The results of the localization of FAK and YAP in the chondrocytes cultured in different viscoelastic hydrogels, indicates that the activity of FAK and YAP mechanosensitive signal molecules are influenced by the viscoelastic properties (Figures 7, 8). Faster stress relaxation can promote the increased localization of FAK and YAP around the nucleus in the chondrocytes. Furthermore, the lower level of IL-1β is detected in the faster relaxing hydrogel, suggesting that faster stress relaxation possibly decreases the secretion of IL-1β by chondrocytes (Figure 9). This result can be attributed that faster stress relaxation promotes the growth of chondrocytes, thereby avoiding osteoarthritis (Lima et al., 2007; Klein et al., 2010).

**FIGURE 10:**
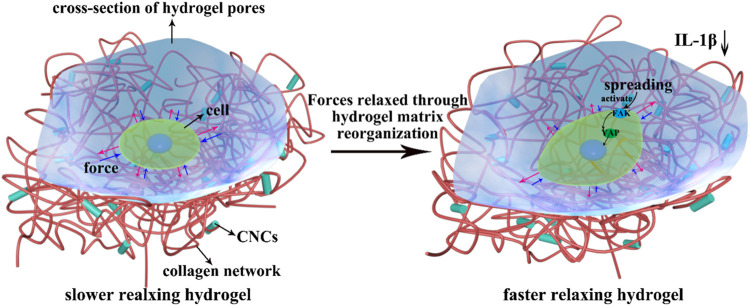
Overview of CNC/COL hydrogel stress relaxation impacting chondrocyte behavior through the mechano-transduction of FAK and YAP. Red arrow represents the force exerted by chondrocytes and blue arrow represents the resisting force applied by hydrogel substrate.

Based on the above results, we proposed a possible molecular mechanism by which the rapid stress relaxation of 2D CNC/COL hydrogel microenvironment regulates chondrocyte behavior (Figure 10). On the surface of hydrogel pore wall where the stress relaxes slowly, the force exerted by the pore wall resists the change of chondrocyte shape over long times (Lee et al., 2017). While on the surface of hydrogel pore wall where the stress relaxes fast, the force can be relaxed or dissipated by the rearrangement of CNC/COL networks over short times so that contributes to the change of chondrocyte shape (Richardson et al., 2018). The dynamically and cyclically mechanical stimuli from the pore wall is continuously fed into the chondrocytes by actin cytoskeleton to activate FAK and YAP signal molecules and impact their activity (Mitra and Schlaepfer, 2006; Sirio et al., 2011; Brusatin et al., 2018). Further, faster mechanical stimuli facilitate the accumulation and translocation of signal molecules around the nucleus. In brief, the force interaction between developing chondrocytes and rapid relaxing CNC/COL hydrogel pore wall transduces and transforms the extracellular mechanical signals into the intracellular biochemical signals, finally facilitating the processes of chondrocytes spreading, shape change, proliferation, and matrix deposition over long timescales. Therefore, we point out FAK and YAP, as mechano-transduction signal molecules, participate in the growth of chondrocytes cultured on the 2D microenvironment constructed by a fast-relaxing CNC/COL hydrogel.

## Conclusion

This work investigates the effects of viscoelastic properties of microporous CNC/COL hydrogel on chondrocyte behaviors and the related intracellular mechano-transduction mechanism. The CNC/COL hydrogels have tuneable viscoelastic properties by changing CNC concentration. The cell aspect ratio, proliferation and levels of COL II and GAGs are enhanced in the hydrogel with faster stress relaxation and creep rates, higher storage modulus and loss modulus. Particularly, the stress relaxation has the strongest correlation with the behavior of chondrocytes, and its authority weight value is as high as 62.547%. In addition, the level of IL-1β, a major driver of osteoarthritis progression, is decreased in faster relaxing hydrogel. Further, FAK and YAP play important roles in the chondrocytes’ responses to the rapid relaxing hydrogel. This study contributes to understanding of how hydrogel viscoelastic properties impact chondrocyte behaviors and implicates stress relaxation as an important design parameter for preparing cartilage scaffolds.

## Data Availability

The original contributions presented in the study are included in the article/Supplementary Material; further inquiries can be directed to the corresponding authors.

## References

[B1] Abitbol T. Kam D. Levi-Kalisman Y. Gray D. G. Shoseyov O. (2018). Surface charge influence on the phase separation and viscosity of cellulose nanocrystals. Langmuir 34, 3925–3933. 10.1021/acs.langmuir.7b04127 PubMed Abstract | 10.1021/acs.langmuir.7b04127 | Google Scholar 29513998

[B2] Andrea G. L. Xiomara F. G. Ferran V. M. Castano A. G. Samitier J. Ramon-Azcon J. (2018). Composite biomaterials as long-lasting scaffolds for 3D bioprinting of highly aligned muscle tissue. Macromol. Biosci. 18, 1800167. 10.1002/mabi.201800167 10.1002/mabi.201800167 | Google Scholar 30156756

[B3] Bauer A. Gu L. Kwee B. Li W. A. Dellacherie M. Celiz A. D. (2017). Hydrogel substrate stress-relaxation regulates the spreading and proliferation of mouse myoblasts. Acta Biomater. 62, 82–90. 10.1016/j.actbio.2017.08.041 PubMed Abstract | 10.1016/j.actbio.2017.08.041 | Google Scholar 28864249 PMC5641979

[B4] Biswal T. BadJena S. K. Pradhan D. (2020). Sustainable biomaterials and their applications: A short review. Mat. Today Proc. 30, 274–282. 10.1016/j.matpr.2020.01.437 10.1016/j.matpr.2020.01.437 | Google Scholar

[B5] Brusatin G. Panciera T. Gandin A. Citron A. Piccolo S. (2018). Biomaterials and engineered microenvironments to control YAP/TAZ-dependent cell behaviour. Nat. Mat. 17, 1063–1075. 10.1038/s41563-018-0180-8 10.1038/s41563-018-0180-8 | Google Scholar PMC6992423 30374202

[B6] Burdick J. A. Prestwich G. D. (2011). Hyaluronic acid hydrogels for biomedical applications. Adv. Mat. 23, H41–H56. H41–H56. 10.1002/adma.201003963 10.1002/adma.201003963 | Google Scholar PMC3730855 21394792

[B7] Cameron A. R. Frith J. E. Cooper-White J. J. (2011). The influence of substrate creep on mesenchymal stem cell behaviour and phenotype. Biomaterials 32, 5979–5993. 10.1016/j.biomaterials.2011.04.003 PubMed Abstract | 10.1016/j.biomaterials.2011.04.003 | Google Scholar 21621838

[B8] Cameron A. R. Frith J. E. Gomez G. A. Yap A. S. Cooper-White J. J. (2014). The effect of time dependent deformation of viscoelastic hydrogels on myogenic induction and Rac1 activity in mesenchymal stem cells. Biomaterials 35, 1857–1868. 10.1016/j.biomaterials.2013.11.023 PubMed Abstract | 10.1016/j.biomaterials.2013.11.023 | Google Scholar 24331708

[B9] Chaudhuri O. Gu L. Klumpers D. Darnell M. Bencherif S. A. Weaver J. C. (2016). Hydrogels with tunable stress relaxation regulate stem cell fate and activity. Nat. Mat. 15, 326–334. 10.1038/nmat4489 10.1038/nmat4489 | Google Scholar PMC4767627 26618884

[B10] Chester D. Kathard R. Nortey J. Nellenbach K. Brown A. C. (2018). Viscoelastic properties of microgel thin films control fibroblast modes of migration and pro-fibrotic responses. Biomaterials 185, 371–382. 10.1016/j.biomaterials.2018.09.012 PubMed Abstract | 10.1016/j.biomaterials.2018.09.012 | Google Scholar 30292092

[B11] Daheshia M. Yao J. Q. (2008). The interleukin 1β pathway in the pathogenesis of osteoarthritis. J. Rheumatol. 35, 2306–2312. 10.3899/jrheum.080346 PubMed Abstract | 10.3899/jrheum.080346 | Google Scholar 18925684

[B12] Discher D. E. Janmey P. Wang Y. (2005). Tissue cells feel and respond to the stiffness of their substrate. Science 310, 1139–1143. 10.1126/science.1116995 PubMed Abstract | 10.1126/science.1116995 | Google Scholar 16293750

[B13] Engler A. J. Sen S. Sweeney H. L. Discher D. E. (2006). Matrix elasticity directs stem cell lineage specification. Cell 126, 677–689. 10.1016/j.cell.2006.06.044 PubMed Abstract | 10.1016/j.cell.2006.06.044 | Google Scholar 16923388

[B14] Getgood A. Brooks R. Fortier L. Rushton N. (2009). Articular cartilage tissue engineering: today's research, tomorrow's practice? J. Bone Jt. Surg. Br. volume 91, 565–576. 10.1302/0301-620x.91b5.21832 10.1302/0301-620x.91b5.21832 | Google Scholar 19407287

[B15] Jayaramudu T. Ko H. U. Kim H. C. Kim J. Muthoka R. Kim J. (2018). Electroactive hydrogels made with polyvinyl alcohol/cellulose nanocrystals. Materials 11 (9), 1615. 10.3390/ma11091615 10.3390/ma11091615 | Google Scholar PMC6163614 30181521

[B16] Jayaramudu T. Ko H. U. Kim H. C. Kim J. W. Kim J. (2019). Swelling behavior of polyacrylamide–cellulose nanocrystal hydrogels: swelling kinetics, temperature, and pH effects. Materials 12 (13), 2080. 10.3390/ma12132080 10.3390/ma12132080 | Google Scholar PMC6650916 31261618

[B17] Klein T. J. Rizzi S. C. Schrobback K. Reichert J. C. Jeon J. E. Crawford R. W. (2010). Long-term effects of hydrogel properties on human chondrocyte behavior. Soft Matter 6, 5175. 10.1039/c0sm00229a 10.1039/c0sm00229a | Google Scholar

[B18] Lachowski D. Cortes E. Robinson B. Rice A. Rombouts K. Del Rio Hernandez A. E. (2017). Fak controls the mechanical activation of yap, a transcriptional regulator required for durotaxis. FASEB J. 32, 1099–1107. 10.1096/fj.201700721r 10.1096/fj.201700721r | Google Scholar 29070586

[B19] Lee C. D. Gleghorn J. P. Choi N. W. Cabodi M. Stroock A. D. Bonassar L. J. (2007). Integration of layered chondrocyte-seeded alginate hydrogel scaffolds. Biomaterials 28, 2987–2993. 10.1016/j.biomaterials.2007.02.035 PubMed Abstract | 10.1016/j.biomaterials.2007.02.035 | Google Scholar 17382380

[B20] Lee H. P. Gu L. Mooney D. J. Levenston M. E. Chaudhuri O. (2017). Mechanical confinement regulates cartilage matrix formation by chondrocytes. Nat. Mat. 16, 1243–1251. 10.1038/nmat4993 10.1038/nmat4993 | Google Scholar PMC5701824 28967913

[B21] Lee S. Stanton A. E. Tong X. Yang F. (2019). Hydrogels with enhanced protein conjugation efficiency reveal stiffness-induced yap localization in stem cells depends on biochemical cues. Biomaterials 202, 26–34. 10.1016/j.biomaterials.2019.02.021 PubMed Abstract | 10.1016/j.biomaterials.2019.02.021 | Google Scholar 30826537 PMC6447317

[B22] Lima E. G. Bian L. Ng K. W. Mauck R. Byers B. Tuan R. (2007). The beneficial effect of delayed compressive loading on tissue-engineered cartilage constructs cultured with TGF-β3. Osteoarthr. Cartil. 15, 1025–1033. 10.1016/j.joca.2007.03.008 10.1016/j.joca.2007.03.008 | Google Scholar PMC2724596 17498976

[B23] Liu D. L. Chen Z. B. Du X. Y. Liu Z. (2017). Study of structural parameters on the adsorption selectivity of a molecularly imprinted polymer. J. Macromol. Sci. Part A 54, 622–628. 10.1080/10601325.2017.1316670 10.1080/10601325.2017.1316670 | Google Scholar

[B24] Liu D. L. Dong X. F. Han B. G. Huang H. Qi M. (2020). Cellulose nanocrystal/collagen hydrogels reinforced by anisotropic structure: Shear viscoelasticity and related strengthening mechanism. Compos. Commun. 21, 100374. 10.1016/j.coco.2020.100374 10.1016/j.coco.2020.100374 | Google Scholar

[B25] Liu D. L. Dong X. F. Liu H. Y. Zhao Y. Qi M. (2021). Effect of pore orientation on shear viscoelasticity of cellulose nanocrystal/collagen hydrogels. J. Appl. Polym. Sci. 138 (7), e49856. 10.1002/app.49856 10.1002/app.49856 | Google Scholar

[B26] Loebel C. Kwon M. Y. Wang C. Han L. Mauck R. L. Burdick J. A. (2020). Metabolic labeling to probe the spatiotemporal accumulation of matrix at the chondrocyte-hydrogel interface. Adv. Funct. Mat. 30, 1909802. 10.1002/adfm.201909802 10.1002/adfm.201909802 | Google Scholar PMC8240476 34211359

[B27] Lou J. Z. Stowers R. Nam S. Xia Y. Chaudhuri O. (2018). Stress relaxing hyaluronic acid-collagen hydrogels promote cell spreading, fiber remodeling, and focal adhesion formation in 3D cell culture. Biomaterials 154, 213–222. 10.1016/j.biomaterials.2017.11.004 PubMed Abstract | 10.1016/j.biomaterials.2017.11.004 | Google Scholar 29132046

[B28] Lu X. L. Mow V. C. (2008). Biomechanics of articular cartilage and determination of material properties. Med. Sci. Sports Exerc. 40, 193–199. 10.1249/mss.0b013e31815cb1fc PubMed Abstract | 10.1249/mss.0b013e31815cb1fc | Google Scholar 18202585

[B29] Martha A. H. Mathew A. P. Oksman] K. (2014). Gas permeability and selectivity of cellulose nanocrystals films (layers) deposited by spin coating. Carbohydr. Polym. 112, 494–501. 10.1016/j.carbpol.2014.06.036 PubMed Abstract | 10.1016/j.carbpol.2014.06.036 | Google Scholar 25129773

[B30] Matellan C. Hernandez A. E. (2019). Engineering the cellular mechanical microenvironment-from bulk mechanics to the nanoscale. J. Cell Sci. 132, jcs229013. 10.1242/jcs.229013 PubMed Abstract | 10.1242/jcs.229013 | Google Scholar 31040223

[B31] Mauck R. L. Soltz M. A. Wang C. B. Wong D. D. Chao P. H. G. Valhmu W. B. (2000). Functional tissue engineering of articular cartilage through dynamic loading of chondrocyte-seeded agarose gels. J. Biomech. Eng. 122, 252–260. 10.1115/1.429656 PubMed Abstract | 10.1115/1.429656 | Google Scholar 10923293

[B32] McKinnon D. D. Domaille D. W. Cha J. N. Anseth K. S. (2013). Biophysically defined and cytocompatible covalently adaptable networks as viscoelastic 3D cell culture systems. Adv. Mat. 26, 865–872. 10.1002/adma.201303680 10.1002/adma.201303680 | Google Scholar PMC4582033 24127293

[B33] Mitra S. K. Schlaepfer D. D. (2006). Integrin-regulated FAK-Src signaling in normal and cancer cells. Curr. Opin. Cell Biol. 18, 516–523. 10.1016/j.ceb.2006.08.011 PubMed Abstract | 10.1016/j.ceb.2006.08.011 | Google Scholar 16919435

[B34] Mow V. C. Ratcliffe A. Poole A. R. (1991). Cartilage and diarthrodial joints as paradigms for hierarchical materials and structures. Biomaterials 13, 67–97. 10.1016/0142-9612(92)90001-5 10.1016/0142-9612(92)90001-5 | Google Scholar 1550898

[B35] Patel J. M. Loebel C. Saleh K. S. Wise B. C. Bonnevie E. D. Miller L. M. (2021). Stabilization of damaged articular cartilage with hydrogel-mediated reinforcement and sealing. Adv. Healthc. Mat. 10, e2100315. 10.1002/adhm.202100315 10.1002/adhm.202100315 | Google Scholar PMC8224478 33738988

[B36] Pelletier J. P. DiBattista J. A. Roughley P. McCollum R. Martel-Pelletier J. (1993). Cytokines and inflammation in cartilage degradation. Rheumatic Dis. Clin. N. Am. 19, 545–568. 10.1016/s0889-857x(21)00331-8 10.1016/s0889-857x(21)00331-8 | Google Scholar 8210574

[B37] Richardson B. M. Wilcox D. G. Randolph M. A. Anseth K. S. (2018). Hydrazone covalent adaptable networks modulate extracellular matrix deposition for cartilage tissue engineering. Acta. Biomater., 83. 71–82. 10.1016/j.actbio.2018.11.014 PubMed Abstract | 10.1016/j.actbio.2018.11.014 | Google Scholar 30419278 PMC6291351

[B38] Sirio D. Leonardo M. Mariaceleste A. Enzo E. Giulitti S. Cordenonsi M. (2011). Role of YAP/TAZ in mechanotransduction. Nature 474, 179–183. 10.1038/nature10137 PubMed Abstract | 10.1038/nature10137 | Google Scholar 21654799

[B39] Solon J. Levental I. Sengupta K. Georges P. C. Janmey P. A. (2007). Fibroblast adaptation and stiffness matching to soft elastic substrates. Biophys. J. 93, 4453–4461. 10.1529/biophysj.106.101386 PubMed Abstract | 10.1529/biophysj.106.101386 | Google Scholar 18045965 PMC2098710

[B40] Trache D. Hussin M. H. Haafiz M. Thakur V. K. (2017). Recent progress in cellulose nanocrystals: sources and production. Nanoscale 9, 1763–1786. 10.1039/c6nr09494e PubMed Abstract | 10.1039/c6nr09494e | Google Scholar 28116390

[B41] Walker C. J. Crocini C. Ramirez D. Anouk R. K. Joseph C. G. Brian A. A. (2021). Author correction: nuclear mechanosensing drives chromatin remodeling in persistently activated fibroblasts. Nat. Biomed. Eng. 5, 1517–1518. 10.1038/s41551-021-00748-3 PubMed Abstract | 10.1038/s41551-021-00748-3 | Google Scholar 34050334

[B42] Yang W. Zheng Y. Y. Chen J. Zhu Q. Feng L. Lan Y. (2019). Preparation and characterization of the collagen/cellulose nanocrystals/USPIO scaffolds loaded kartogenin for cartilage regeneration. Mater. Sci. Eng. C 99, 1362–1373. 10.1016/j.msec.2019.02.071 10.1016/j.msec.2019.02.071 | Google Scholar 30889670

[B43] Zhang Q. Lu H. X. Kawazoe N. Chen G. (2014). Pore size effect of collagen scaffolds on cartilage regeneration. Acta Biomater. 10, 2005–2013. 10.1016/j.actbio.2013.12.042 PubMed Abstract | 10.1016/j.actbio.2013.12.042 | Google Scholar 24384122

